# Effects of paroxetine combined with sulpiride on sleep status and quality of life in patients with depressive episodes

**DOI:** 10.3389/fpsyt.2025.1600675

**Published:** 2025-09-17

**Authors:** Di Zhao, Chunyu Liu

**Affiliations:** ^1^ Depression Section, The First Psychiatric Hospital Of Harbin, Harbin, Heilongjiang, China; ^2^ Internal Medicine, Wanjia Compulsory Isolation and Drug Rehabilitation Hospital in Harbin, Harbin, Heilongjiang, China

**Keywords:** paroxetine, sulpiride, depressive disorder, sleep status, quality of life

## Abstract

**Objective:**

Assess the clinical efficacy of paroxetine and sulpiride IV drip in treating depression and its impact on sleep and quality of life.

**Methods:**

Retrospective analysis of 80 depression patients, divided into a study group treated with paroxetine and sulpiride IV drip, and a control group treated with paroxetine alone, over 8 weeks. HAMD and SF-36 assessed depression and quality of life; TESS evaluated adverse reactions.

**Results:**

HAMD scores decreased in both groups, with the study group showing better depression status at 1, 2, and 4 weeks (P<0.05) and higher effective rates (P<0.05). The study group had faster symptom relief. SF-36 scores in role physical, general health, vitality, and role emotional were higher in the study group (P<0.05). TESS scores showed no significant difference between groups (P>0.05), with mild adverse reactions.

**Conclusion:**

Paroxetine with sulpiride IV drip offers rapid relief and high safety in depression treatment, improving sleep and quality of life.

## 1 Introduction

Depressive Disorder is a common, chronic mental disorder characterized by a depressed mood (such as feelings of sadness, irritability, or emptiness) and anhedonia (such as a decreased ability to feel pleasure or enjoyment in daily activities). People with depression also suffer from a variety of other symptoms, which can be divided into cognitive symptoms, emotional symptoms, and vegetative symptoms (1). According to official statistics in 2021, there will be more than 350 million depressed patients worldwide. The number of depressed patients in China is as high as 54 million, accounting for 4.2% of the total population of China. In other words, there are 4 depressed patients in 1,000 people. Depression is highly recurrent. According to incomplete statistics, the annual recurrence rate of outpatient depression patients is about 40% (2). Severe depression has a high disability rate. Patients are depressed, pessimistic, and world-weary. Their social functions are significantly reduced and they are unable to play social roles. Some even self-harm or commit suicide, which brings pain and economic burden to their families. For the country, the treatment costs of patients with depression also put a lot of pressure on medical insurance.

The etiology and pathological mechanism of depression are complex and may involve multiple aspects, but there is no definitive research yet. It may be a disease caused by biological, psychological, and social factors (3, 4). In clinical work, the author found that depression has a high heritability and the concomitant rate of relatives of patients with depression is higher (5, 6). Family studies have shown that the heritability of depression is about 37%, the concomitant rate of identical twins is about 50%, and the concomitant rate of fraternal twins is about 10% to 25% (7). At present, there is no definite conclusion on the pathological mechanism of depression. There are mainly several hypotheses, such as the hypothalamus-pituitary-adrenal (HPA) axis hyperfunction hypothesis, the neurodegeneration hypothesis, etc. (8), among which the most concerned are the monoamine neurotransmitter hypothesis and the HPA axis hyperfunction hypothesis (9–11). The monoamine neurotransmitter hypothesis believes that the reduced activity of dopamine (DA), 5-hydroxytryptamine (5-HT), and norepinephrine is related to the occurrence of depression (12). The HPA axis is the center of homeostasis, stress response, energy metabolism, and neuropsychiatric function. Studies have found that the HPA axis is hyperfunctional in patients with depression.

Insomnia is the most common physical symptom of depression. An epidemiological study in Mexico suggests that about 80% of depressed patients suffer from insomnia (13). Chronic insomnia patients should be alert to the occurrence of depression (14). Insomnia may aggravate depressive symptoms, increase the difficulty of depression treatment, reduce the quality of life of depressed patients, and increase the risk of suicide in depressed patients (15). Studies have found that the severity of insomnia symptoms can be used as an evaluation factor for the prognosis of depression (16). The more severe the accompanying insomnia symptoms, the worse the prognosis of depression. Depressed patients with severe insomnia often respond poorly to treatment. Insomnia also increases the risk of relapse of depression (17). Clinically, some depressed patients have residual insomnia after their depressive symptoms improve. Studies have found that residual insomnia symptoms are positively correlated with the recurrence of depression (18). Treatment of depressive insomnia is an important part of depression treatment. Improvement of depressive insomnia helps promote the remission of depression and is an important indicator for evaluating the efficacy of antidepressant treatment. How to effectively treat depressive insomnia has become a hot topic in the field of depression research.

For the treatment of depressive insomnia, both China and foreign countries currently advocate that depression should be treated as the main treatment. First, antidepressants with sleep-improving effects should be selected. If necessary, antidepressants and hypnotics can be used in combination (19). Some antidepressants can improve insomnia symptoms while improving depressive mood, such as paroxetine, amitriptyline, doxepin, mirtazapine, etc. Among them, paroxetine has an outstanding antidepressant effect. It is the most potent drug in the SSRIs that inhibits N5-HT reuptake and is one of the most commonly used antidepressants. Paroxetine is also the drug with the greatest impact on sleep structure among SSRIs, improving patients’ satisfaction with sleep (20). In addition, sulpiride is a commonly used drug in clinical psychiatry. It can block patients’ DA2 receptors (dopamine) and reduce the activity of other transmitters in the brain. Patients will not experience obvious anti-excitatory effects after using it. It can effectively improve patients’ symptoms such as inferiority, slow thinking, despair, and anxiety (21). In addition, sulpiride has a low effect on extrapyramidal symptoms and can also inhibit gastric acid secretion, thereby reducing adverse reactions. However, antidepressants have many side effects, which can cause adverse reactions in multiple systems such as cardiovascular, nervous, digestive, and reproductive systems, especially after long-term use, which limits the application of antidepressants (22). For patients with other mental or physical diseases, especially elderly patients, the choice of antidepressants is more difficult.

In general, it is very important to relieve the symptoms of depression patients as soon as possible and reduce the risk of suicide. This study explored the efficacy and safety of paroxetine combined with sulpiride in the treatment of patients with depression and analyzed the effects on their sleep status and quality of life.

## 2 Methods and materials

### 2.1 Basic information

A retrospective analysis was performed on 80 patients with depression admitted to our hospital from January 2022 to January 2024. The patients were divided into two groups (40 cases in each group) according to different treatment methods.

Inclusion criteria: (1) met the diagnostic criteria for depressive episodes in the 10th edition of the International Classification of Disease (ICD-10); (2) aged 18 to 60 years; (3) had not received any antidepressant treatment within 1 month before enrollment; (4) had a HAMD score ≥ 18 points.

Exclusion criteria: (1) patients with severe physical illness and organic brain disease; (2) patients with bipolar disorder; (3) patients with alcohol or drug dependence; (4) patients during pregnancy and lactation; (5) patients with psychotic symptoms; (6) patients with drug addiction or dependence; (7) patients with language, hearing or cognitive dysfunction.

This study was approved by the Ethics Committee of The First Psychiatric Hospital Of Harbin.

### 2.2 Treatment

Both groups received standardized psychological interventions delivered by certified psychotherapists (with ≥5 years of clinical experience). The interventions included: cognitive behavioral therapy (CBT): 60-minute individual sessions, conducted biweekly for 8 weeks, focusing on cognitive restructuring and behavioral activation; supportive therapy: 30-minute individual sessions, weekly for 8 weeks, emphasizing emotional validation and coping skill training; psychoanalytic therapy: 45-minute individual sessions, once every 2 weeks for 8 weeks, exploring unconscious conflicts and interpersonal patterns. Adherence was monitored via session attendance records (≥80% attendance defined as good adherence), with no significant difference in adherence rates between the study group (92.5%) and control group (90.0%) (χ²=0.183, P=0.669).

The study group was given oral paroxetine (specification: 20 mg/tablet) for treatment: initial dose 20 mg/d (1 tablet/d) for the first 2 weeks, and adjusted to 20–40 mg/d (1–2 tablets/d) from week 3 to week 8 based on clinical response, with a mean maintenance dose of 28.5 ± 6.3 mg/d. At the same time, sulpiride was used with an intravenous drip in the first 2 weeks of treatment (sulpiride injection of 0.1 g added to 250 ml of normal saline, once a day). The control group was treated with oral paroxetine using the same dosing strategy: initial dose 20 mg/d (1 tablet/d) for the first 2 weeks, adjusted to 20–40 mg/d (1–2 tablets/d) from week 3 to week 8, with a mean maintenance dose of 27.8 ± 5.9 mg/d, and normal saline was given intravenously (250 ml, once a day) for the first 2 weeks of treatment. Statistical analysis showed no significant difference in mean paroxetine maintenance dose between the two groups (t=0.523, P=0.602). The treatment time for both groups was 8 weeks, during which other antidepressants, mood stabilizers, and antipsychotics were prohibited, and low-dose benzodiazepines could be temporarily used in combination.

### 2.3 Observation indicators

1. Depression: HAMD was used to assess the depression of the patients. Two trained psychiatrists assessed the depression of the patients before treatment, 1 week after treatment, 2 weeks after treatment, 4 weeks after treatment, and 8 weeks after treatment. The scale has 17 items, ranging from none to extremely severe, with scores of 0-4. The scoring criteria are: a total score of <7 is normal, 7–16 is possible depression, 17–23 is definite depression, and 24 or more is severe depression. The efficacy evaluation is based on a HAMD score reduction rate of ≥75% for remission, ≥50% for marked effect, ≥25% for improvement, and <25% for ineffectiveness (11, 15).
2. Quality of life: The MOS item short from the health survey (SF-36) was used to assess the patient’s health-related quality of life. The questionnaire includes physical functioning (PF), body pain (BP), role physical (RP), general health (GH), vitality (VT), social functioning (SF), mental health (MH), and role emotional (RE). The patient’s score was converted into a standard score. The higher the score, the less functional impairment and the better the quality of life.
3. Sleep status: The Pittsburgh Sleep Quality Index (PSQI) was used to assess sleep quality. This 19-item scale evaluates sleep duration, sleep latency, sleep efficiency, sleep disturbances, use of sleeping medications, and daytime dysfunction over the past month. Total scores range from 0 to 21, with higher scores indicating poorer sleep quality. Assessments were conducted at baseline, 1week, 2 weeks, 4 weeks, and 8 weeks after treatment.
4. Adverse reactions: The adverse reactions scale (TESS) is used to evaluate the adverse reactions of patients. The TESS scale includes 34 symptoms, and each symptom is scored on a 0–4 rating scale. The score range is 0–136 points. The higher the score, the more severe the adverse reaction.

### 2.4 Statistical method

Data were analyzed using IBM SPSS 26.0. Normality of continuous variables was assessed using the Shapiro-Wilk test and Q-Q plots; variables following a normal distribution were described by mean ± standard deviation, while non-normally distributed variables were expressed as median (interquartile range) and analyzed using Mann-Whitney U tests. Categorical variables were described by frequency and percentage and analyzed using chi-square tests. For longitudinal data (e.g., HAMD and PSQI scores at multiple time points), repeated measures ANOVA with Bonferroni correction for multiple comparisons was applied to account for within-subject changes and reduce Type I error risk. Two independent sample t-tests were used for between-group comparisons of single time-point parameters, with Bonferroni correction applied for multiple dimensions (e.g., SF-36 subscales). Sample size was determined based on preliminary data indicating a minimum of 36 patients per group to detect a medium effect size (d=0.5) with 80% power at α=0.05. Statistical significance was defined as P<0.05.

## 3 Results

### 3.1 Patient baseline data

Patients were divided into the study group (n=40) and control group (n=40) based on different treatment methods. The baseline demographic and clinical characteristics of the two groups are shown in the table below, with no statistically significant differences observed (all P>0.05), indicating comparability (**Table 1**).

**Table 1:** Patient baseline data.

Baseline characteristics	Study group (n=40)	Control group (n=40)	χ²/t value	P value
Age (years), x ± s	35.76 ± 9.24	36.68 ± 10.35	0.438	0.662
Gender, n (%)			0.125	0.724
Male	18 (45.0)	16 (40.0)		
Female	22 (55.0)	24 (60.0)		
Disease course (months), x ± s	18.30 ± 8.29	19.51 ± 7.93	0.657	0.512
Baseline HAMD score, x ± s	28.30 ± 3.90	28.78 ± 4.10	0.821	0.413
Depressive subtypes, n (%)			1.023	0.800
Unipolar depression	32 (80.0)	30 (75.0)		
Bipolar depression (depressive episode)	8 (20.0)	10 (25.0)		
Hamilton Anxiety Scale (HAMA) score, x ± s	18.25 ± 4.32	17.86 ± 4.51	0.412	0.681
Sleep disorder types, n (%)			0.756	0.685
Difficulty falling asleep	22 (55.0)	20 (50.0)		
Sleep maintenance disorder	15 (37.5)	18 (45.0)		
Early awakening	3 (7.5)	2 (5.0)		
Pittsburgh Sleep Quality Index (PSQI) score, x ± s	12.36 ± 3.15	12.89 ± 3.27	0.725	0.470

HAMD, Hamilton Depression Rating Scale; HAMA, Hamilton Anxiety Rating Scale; PSQI, Pittsburgh Sleep Quality Index.

### 3.2 Comparison of efficacy

In the study group, 18 cases were remission, 16 cases were markedly effective, 3 cases were improved, and 3 cases were ineffective, with an efficiency rate (remission & markedly effective) of 85.00%; in the control group, 14, 12, 9 and 5 cases respectively, with a marked efficiency rate of 65.00%. A chi-square test was specifically applied to compare the efficiency rates (remission + marked effect) between the two groups (χ²=4.267, P=0.039), confirming that the efficiency rate of the study group was significantly higher than that of the control group (P<0.05). (**Table 2**).

**Table 2:** Comparison of efficacy [n (%)].

Group	Remission	Marked effect	Improvement	Ineffectiveness	Efficiency
study group (n=40)	18 (45.00)	16 (40.00)	3 (7.50)	3 (7.50)	34 (85.00)
control group (n=40)	14 (35.00)	12 (30.00)	9 (22.50)	5 (12.50)	26 (65.00)
χ^2^					4.267
P					0.039

### 3.3 Comparison of HAMD scores before and after treatment

A repeated measures analysis of variance (MANOVA) with Bonferroni correction was applied to analyze longitudinal HAMD score changes, followed by *post-hoc* independent samples t-tests for inter-group comparisons. MANOVA results (Bonferroni-corrected): main effect of time: HAMD scores decreased significantly over 8 weeks in both groups (F=189.623, P<0.001); main effect of group: overall HAMD scores in the study group were lower (F=12.375, P=0.001); Time×Group interaction: score reduction rate differed between groups (F=8.542, P<0.001).

**Figure 1:**
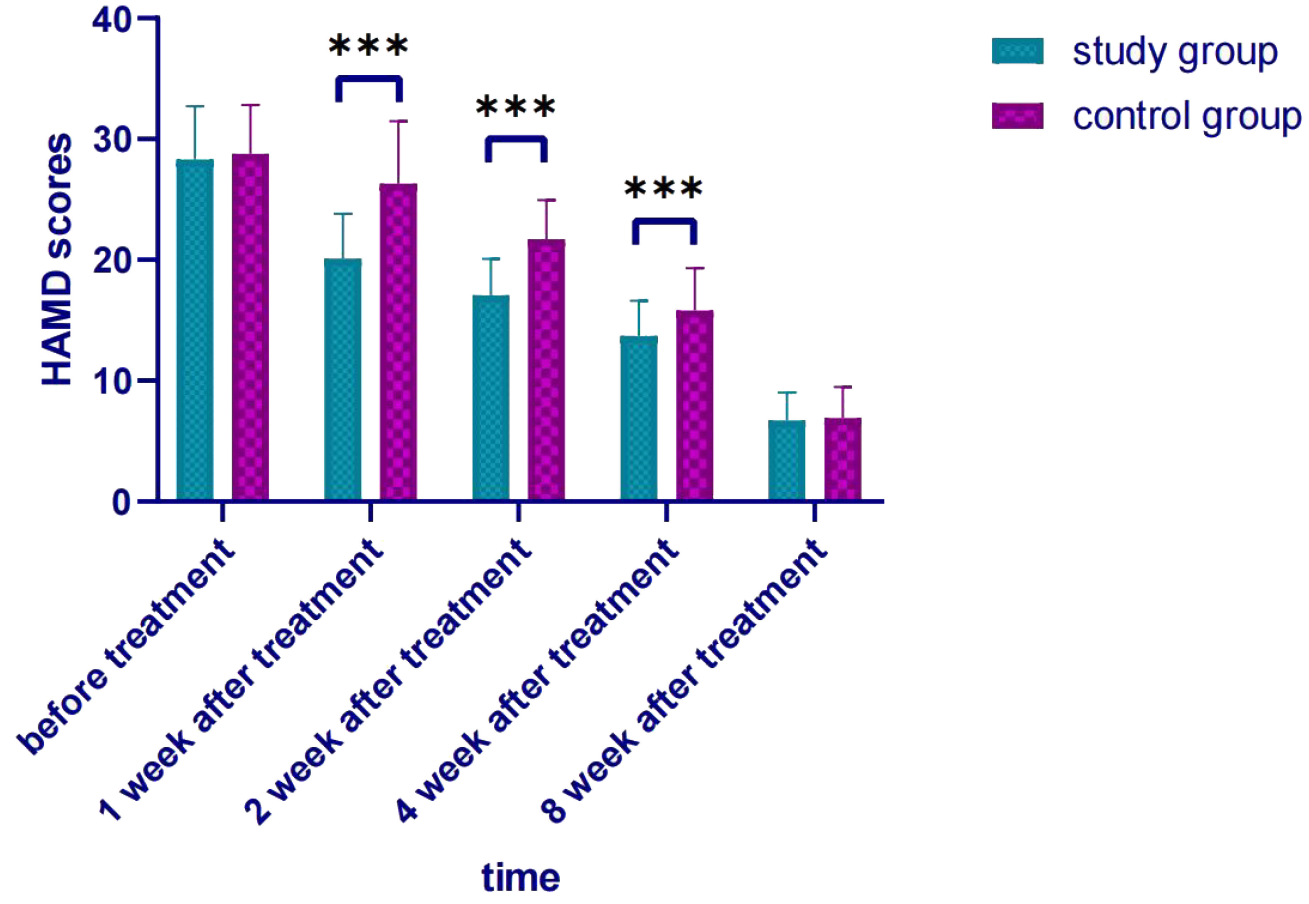
Comparison of HAMD scores after treatment. *** p < 0.001 indicates a highly significant difference between the study group and the control group at the specified time points.

*Post-hoc* comparisons (Bonferroni-corrected): Before treatment, no inter-group difference (t=-0.507, P=0.614); 1 week (t=-6.138, P<0.001), 2 weeks (t=-5.093, P<0.001), 4 weeks (t=-2.936, P=0.004) after treatment, study group HAMD score was significantly lower; 8 weeks after treatment, no inter-group difference (t=-0.312, P=0.756) (**Table 3**, **Figure 1**).

**Table 3:** Comparison of HAMD scores before and after treatment $\left(\bar{x}\pm s\right)$ .

Group	Before treatment	1 week after treatment	2 weeks after treatment	4 weeks after treatment	8 weeks after treatment
study group (n=40)	28.30 ± 4.42	20.13 ± 3.70	18.08 ± 2.99	13.73 ± 2.89	6.73 ± 2.29
control group (n=40)	28.78 ± 4.04	26.30 ± 5.17	21.70 ± 3.24	15.83 ± 3.48	6.90 ± 2.57
t	-0.507	-6.138	-5.093	-2.936	-0.312
P	0.614	<0.001	<0.001	0.004	0.756

### 3.4 Comparison of SF-36 scores before and after treatment

**Figure 2:**
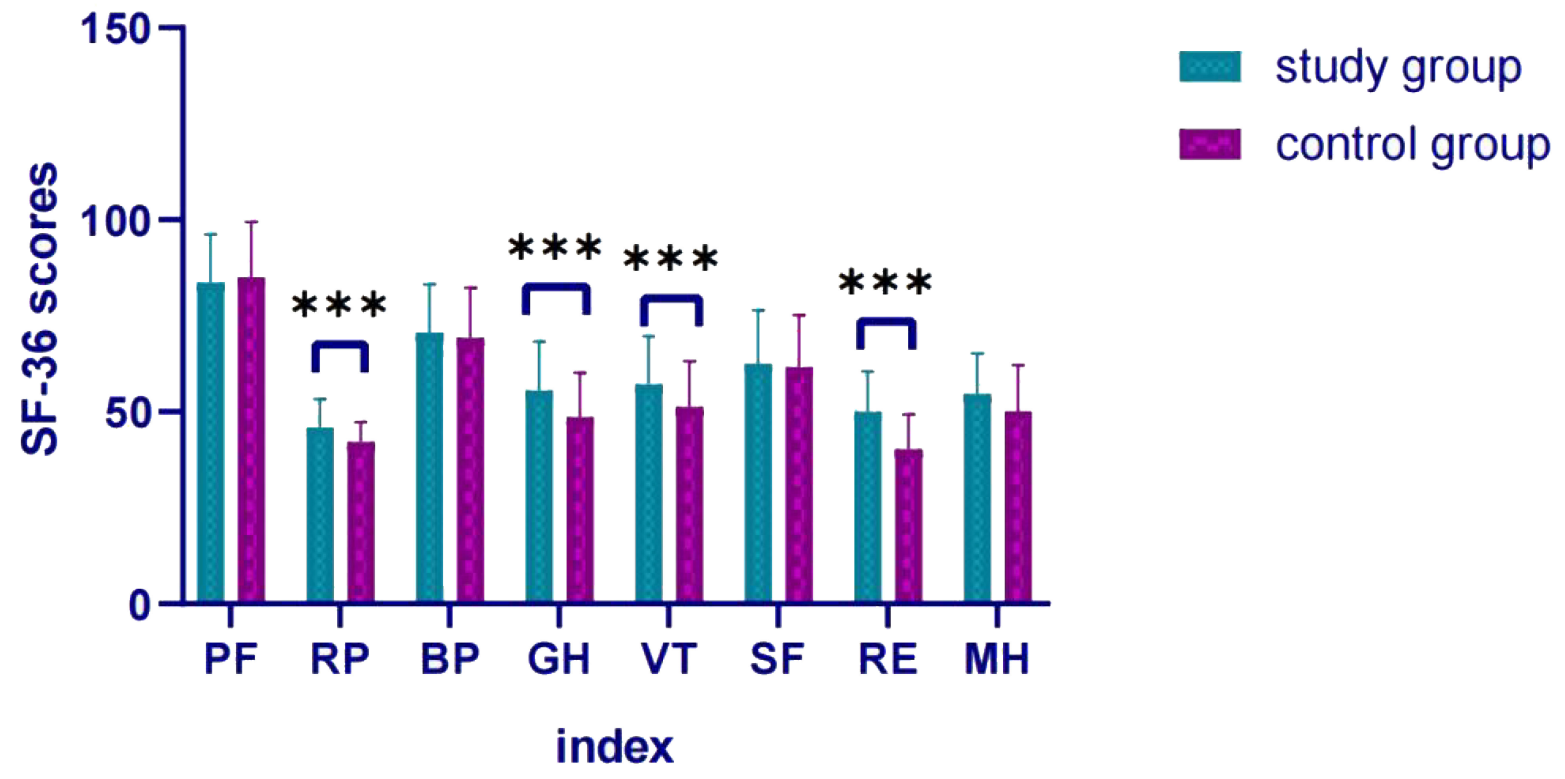
Comparison of SF-36 scores after treatment. *** p < 0.001 indicates a highly significant difference between the study group and the control group at the specified time points.

The SF-36 scores of the study group in the four dimensions of role physical, general health, vitality, and role emotional were higher (all P < 0.05), indicating that the quality of life of the patients in the study group was better after treatment (**Table 4**, **Figure 2**).

**Table 4:** Comparison of SF-36 scores before and after treatment $\left(\bar{x}\pm s\right)$ .

Item	Study group (n=40)	Control group (n=40)	t	P
PF				
before treatment	62.55 ± 11.73	62.10 ± 13.02	0.162	0.871
after treatment	83.65 ± 12.54	85.03 ± 14.43	-0.457	0.649
RP				
before treatment	34.60 ± 6.66	34.95 ± 7.20	-0.226	0.822
after treatment	45.93 ± 7.43	42.15 ± 5.15	2.644	0.010
BP				
before treatment	58.88 ± 13.83	60.18 ± 14.97	0.403	0.688
after treatment	70.65 ± 12.54	69.33 ± 12.97	0.463	0.645
GH				
before treatment	36.20 ± 10.46	35.45 ± 10.20	0.325	0.746
after treatment	55.55 ± 12.73	48.63 ± 11.56	2.545	0.013
VT				
before treatment	34.80 ± 8.67	36.80 ± 8.90	-1.018	0.312
after treatment	57.18 ± 12.56	51.33 ± 11.88	2.140	0.035
SF				
before treatment	48.45 ± 12.55	48.18 ± 13.87	0.091	0.927
after treatment	62.55 ± 13.88	61.70 ± 13.51	0.278	0.782
RE				
before treatment	20.70 ± 7.80	20.38 ± 8.60	0.174	0.862
after treatment	50.05 ± 10.53	40.33 ± 8.92	4.455	<0.001
MH				
before treatment	39.45 ± 7.81	40.33 ± 6.79	-0.538	0.592
after treatment	54.65 ± 10.52	50.23 ± 11.94	1.757	0.083

The questionnaire includes physical functioning (PF), body pain (BP), role physical (RP), general health (GH), vitality (VT), social functioning (SF), mental health (MH), and role emotional (RE).

### 3.5 Comparison of PSQI scores before and after treatment

A repeated measures analysis of variance (MANOVA) with Bonferroni correction was used to analyze longitudinal PSQI score changes, followed by *post-hoc* independent samples t-tests for inter-group comparisons. MANOVA results (Bonferroni-corrected): main effect of time: PSQI scores decreased significantly over 8 weeks in both groups (F=156.892, P<0.001); main effect of group: overall PSQI scores in the study group were lower (F=10.743, P=0.002); Time×Group interaction: score reduction rate differed between groups (F=7.931, P<0.001).

*Post-hoc* comparisons (Bonferroni-corrected): Before treatment, no inter-group difference (t=0.725, P=0.47); 1 week (t=2.943, P=0.004), 2 weeks (t=3.217, P=0.002), 4 weeks (t=4.029, P=0.001), 8 weeks (t=2.874, P=0.005) after treatment, study group PSQI score was significantly lower than control group (**Table 5**).

**Table 5:** Comparison of PSQI scores before and after treatment $\left(\bar{x}\pm s\right)$ .

Time point	Before treatment		1 week after treatment		2 weeks after treatment		4 weeks after treatment		8 weeks after treatment	
Group	Study group	Control group	Study group	Control group	Study group	Control group	Study group	Control group	Study group	Control group
PSQI score (x ± s)	12.36 ± 3.15	12.89 ± 3.27	9.85 ± 2.31	11.67 ± 2.65	8.12 ± 2.56	10.35 ± 2.89	5.78 ± 1.93	8.21 ± 2.34	3.25 ± 1.56	4.89 ± 1.87
t	0.725		2.943		3.217		4.029		2.874	
P	0.47		0.004		0.002		0.001		0.005	

### 3.6 Comparison of TESS scores before and after treatment

The TESS scores of both groups decreased significantly after treatment, with no significant inter-group difference at the same time points (P>0.05). Qualitative analysis showed that the main adverse reactions in the study group included dry mouth (12 cases), dizziness (8 cases), and transient hyperprolactinemia (5 cases); the control group mainly exhibited dry mouth (10 cases), nausea (7 cases), and sexual dysfunction (2 cases). No extrapyramidal symptoms or cardiac adverse events were observed in either group. All reactions were mild and resolved with symptomatic management or dose adjustment (**Table 6**).

**Table 6:** Comparison of TESS scores before and after treatment $\left(\bar{x}\pm s\right)$ .

Group	Before treatment	After treatment
study group (n=40)	41.55 ± 4.43	21.38 ± 2.84
control group (n=40)	41.30 ± 5.03	22.55 ± 3.01
t	0.236	-1.788
P	0.814	0.078

## 4 Discussion

Depression is a common disease in the Department of Psychiatry and Psychology. Some patients have somatic symptoms, and (or) delusions. Depressed patients are depressed and depressed, and their initial depression continues to evolve into grief, inferiority, pain, pessimism, and world-weariness. They feel that living is a burden, negative, escapist, and even have suicidal tendencies and behaviors, which has caused a serious burden on society and family. The treatment effect of some patients is average, especially for patients with recurrent attacks, the treatment effect is worse (23–25). With the changes in life, environment, and other factors, the pressure of social life increases, and sometimes symptoms such as dizziness, chest tightness, limb pain, insomnia, decreased work ability, and other normalized symptoms may not be detected as early signs of depression. About 2/3 of patients think that they have diseases with physical symptoms, which are very likely caused by depression. As the disease progresses, symptoms such as suspicion and anxiety will appear. Negative emotions such as sadness, sorrow, and depression are suppressed and cannot be released, which deteriorates over time and affects normal work and life. With correct treatment in the early stage, patients with depression can integrate well into society and life; but in the middle stage, there are fewer treatments for depression, and the effect is not as fast as that of patients with early depression. Therefore, early identification and treatment are the key to reducing the prevalence of depression.

Sleep problems are common in patients with depression. In recent years, sleep disorders have been listed as one of the diagnostic criteria for depression (26, 27). Previous studies believed that depression causes sleep disorders, that is, the two are a one-way causal relationship. Last few years, a large number of studies have shown that the two have a complex two-way relationship. Residual insomnia symptoms after antidepressant treatment are highly correlated with the recurrence of depression (28). The pathophysiological mechanism of depression has not yet been elucidated. Currently, the most popular theories are the HPA axis hyperactivity theory. The HPA axis hypothesis believes that the HPA axis plays an important role in sleep regulation. If the HPA axis is overactive, it will reduce sleep efficiency and cause sleep disorders. The monoamine neurotransmitter hypothesis believes that 5-HT is one of the causes of sleep disorders in patients with depression. Low levels of 5-HT can cause a recurrence of depression. The characteristic of sleep in patients with depression is the shortened rapid eye movement (REM) sleep latency (29). Scientific research has shown that REM latency is negatively related to the severity of depression, and the latency is consistently prolonged after the condition is relieved, indicating that REM may be able to predict the efficacy of depression treatment. Improving the patient’s sleep can improve the patient’s prognosis (30) and reduce the recurrence rate of the disease.

Paroxetine is a 5-HT reuptake inhibitor and is a commonly used antidepressant drug. It mainly acts on the HPA axis to regulate the secretion of norepinephrine, histamine, etc. in the body, control the patient’s consciousness, and inhibit the reabsorption of 5-HT by the presynaptic membrane of neurons, which can reduce the concentration of 5-HT in the synaptic cleft and promote 5-HT nerve conduction, thereby producing an antidepressant effect (31). In addition, paroxetine can be completely absorbed after oral administration, has a first-pass effect, and is well tolerated by patients (32). Sulpiride is a benzamide antipsychotic drug that can selectively block dopamine D2 receptors and inhibit dopaminergic neurons (33). At the same time, sulpiride also has a blocking effect on D1, D3, and D4 receptors. Taking large amounts of sulpiride will increase the incidence of arrhythmias, so taking small doses can ensure the safety of treatment.

The synergistic efficacy of paroxetine (a selective serotonin reuptake inhibitor, SSRI) and sulpiride (a D_2_ receptor antagonist) in improving depressive symptoms and sleep may be attributed to their complementary modulation of monoaminergic systems and hypothalamic-pituitary-adrenal (HPA) axis function. Paroxetine enhances serotonergic neurotransmission by inhibiting presynaptic 5-HT reuptake, which alleviates core depressive symptoms (e.g., anhedonia, low mood) and regulates sleep-wake cycles via actions on the raphe nuclei (20, 31). This 5-HT elevation also indirectly modulates dopamine (DA) release in the prefrontal cortex, potentially mitigating cognitive deficits associated with depression. Sulpiride, by selectively blocking postsynaptic D_2_ receptors in the mesolimbic pathway, reduces excessive DA turnover in subcortical regions—an effect linked to rapid alleviation of psychic anxiety and psychomotor retardation (21, 33). Importantly, D_2_ antagonism in the hypothalamus may suppress HPA axis hyperactivity by reducing corticotropin-releasing hormone (CRH) secretion, thereby improving sleep continuity and reducing nocturnal awakenings, a key feature of depression-related sleep disturbances (9, 30). Moreover, the short-term intravenous administration of sulpiride ensures rapid attainment of therapeutic concentrations, synergizing with paroxetine’s gradual 5-HT accumulation. This combination may normalize the reciprocal interaction between 5-HT and DA systems: enhanced 5-HT signaling potentiates the D_2_ antagonist-mediated reduction of hyperdopaminergic activity in limbic regions, while sulpiride’s modulation of DA tone may prevent SSRI-induced sexual dysfunction and further stabilize mood (33). Collectively, these mechanisms contribute to accelerated symptom relief and sleep improvement observed in the study group.

The results of this study showed that the combined use of sulpiride intravenous drip was more effective than the single use of antidepressants, and the efficacy was positive (34, 35). This showed that the short-term combined use of sulpiride intravenous drip could quickly improve depressive symptoms, reduce suicidal ideas and behaviors, reduce the risk of suicide, improve patients’ compliance with treatment, and was also beneficial to ward management. In addition, the HAMD scale scores of the study group were lower at 1, 2, and 4 weeks of treatment, indicating that the improvement in compliance was beneficial to improving the effect of drug treatment and alleviating the patient’s depressive symptoms. When the treatment reached the eighth week, the drug dosage of the two groups was consistent, and the scores of depressive symptoms and HAMD scales also tended to be consistent. The SF-36 scores of the study group in the four dimensions of role physical, general health, vitality, and role emotional were all higher than those of the control group, indicating that the patients in the study group had a better quality of life than the control group. Since there was no significant difference in the dimensions of the SF-36 scale between the two groups before treatment, this further reflected that the combined use of sulpiride intravenous drip was more effective than the single use of antidepressants for patients. In addition, no serious adverse reactions occurred in either group in this study, which may be related to the small amount of sulpiride used.

The present study has several limitations. First, the psychological interventions, despite standardized protocols, may have introduced potential bias due to unavoidable variability in therapist adherence to CBT, supportive therapy, and psychoanalytic therapy procedures. Differences in therapist experience, communication styles, or individualized adjustments to intervention content (e.g., emphasis on specific CBT techniques) could have influenced patient outcomes, which were not fully quantified in this study. Second, the retrospective design limits causal inference, as unmeasured confounding factors (e.g., concurrent lifestyle changes) may have affected treatment responses. Third, the 8-week follow-up period is insufficient to evaluate long-term efficacy and relapse rates, particularly for assessing sustained improvements in sleep and quality of life. Finally, the sample size, although justified by power calculation, remains relatively small, which may restrict the generalizability of the findings to broader populations with depression.

In conclusion, short-term paroxetine combined with sulpiride intravenous drip can quickly improve symptoms, improve sleep quality and quality of life, reduce suicide risk, and have high safety, which is worthy of wide clinical application.

## Data Availability

The original contributions presented in the study are included in the article/supplementary material. Further inquiries can be directed to the corresponding author.
